# The BioRECIPE Knowledge Representation Format

**DOI:** 10.1021/acssynbio.4c00096

**Published:** 2024-07-25

**Authors:** Emilee Holtzapple, Gaoxiang Zhou, Haomiao Luo, Difei Tang, Niloofar Arazkhani, Casey Hansen, Cheryl A. Telmer, Natasa Miskov-Zivanov

**Affiliations:** ^†^Electrical and Computer Engineering Department, ^‡^Computational and Systems Biology Department, ^§^Bioengineering Department, University of Pittsburgh, Pittsburgh, Pennsylvania 15260, United States; ∥ Department of Biological Sciences, Carnegie Mellon University, Pittsburgh, Pennsylvania 15213, United States

**Keywords:** modeling, representation format, FAIR principles, signaling pathways, networks, automation

## Abstract

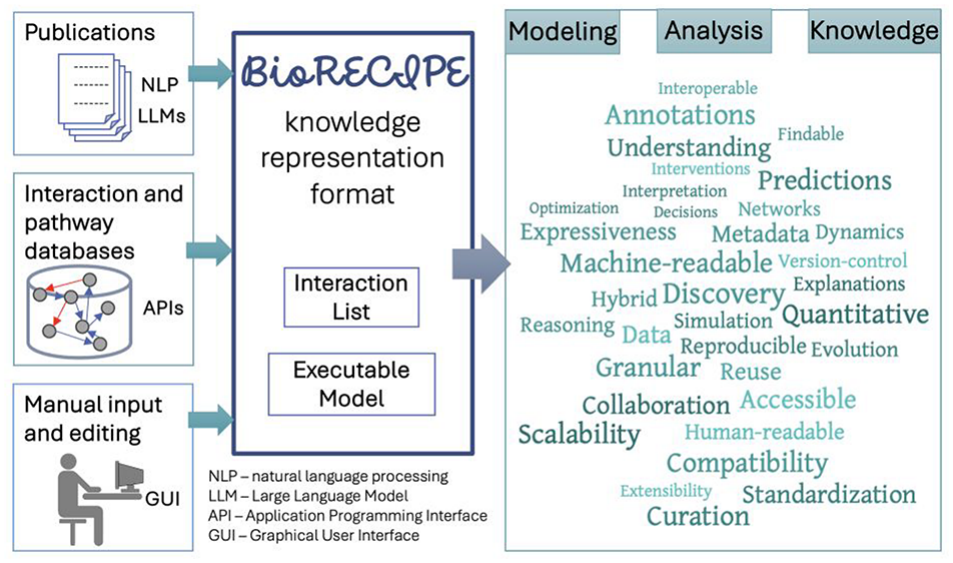

The BioRECIPE (Biological system Representation for Evaluation, Curation, Interoperability, Preserving, and Execution) knowledge representation format was introduced to standardize and facilitate human–machine interaction while creating, verifying, evaluating, curating, and expanding executable models of intra- and intercellular signaling. This format allows a human user to easily preview and modify any model component, while it is at the same time readable by machines and can be processed by a suite of model development and analysis tools. The BioRECIPE format is compatible with multiple representation formats, natural language processing tools, modeling tools, and databases that are used by the systems and synthetic biology communities.

## Introduction

**Figure 1:**
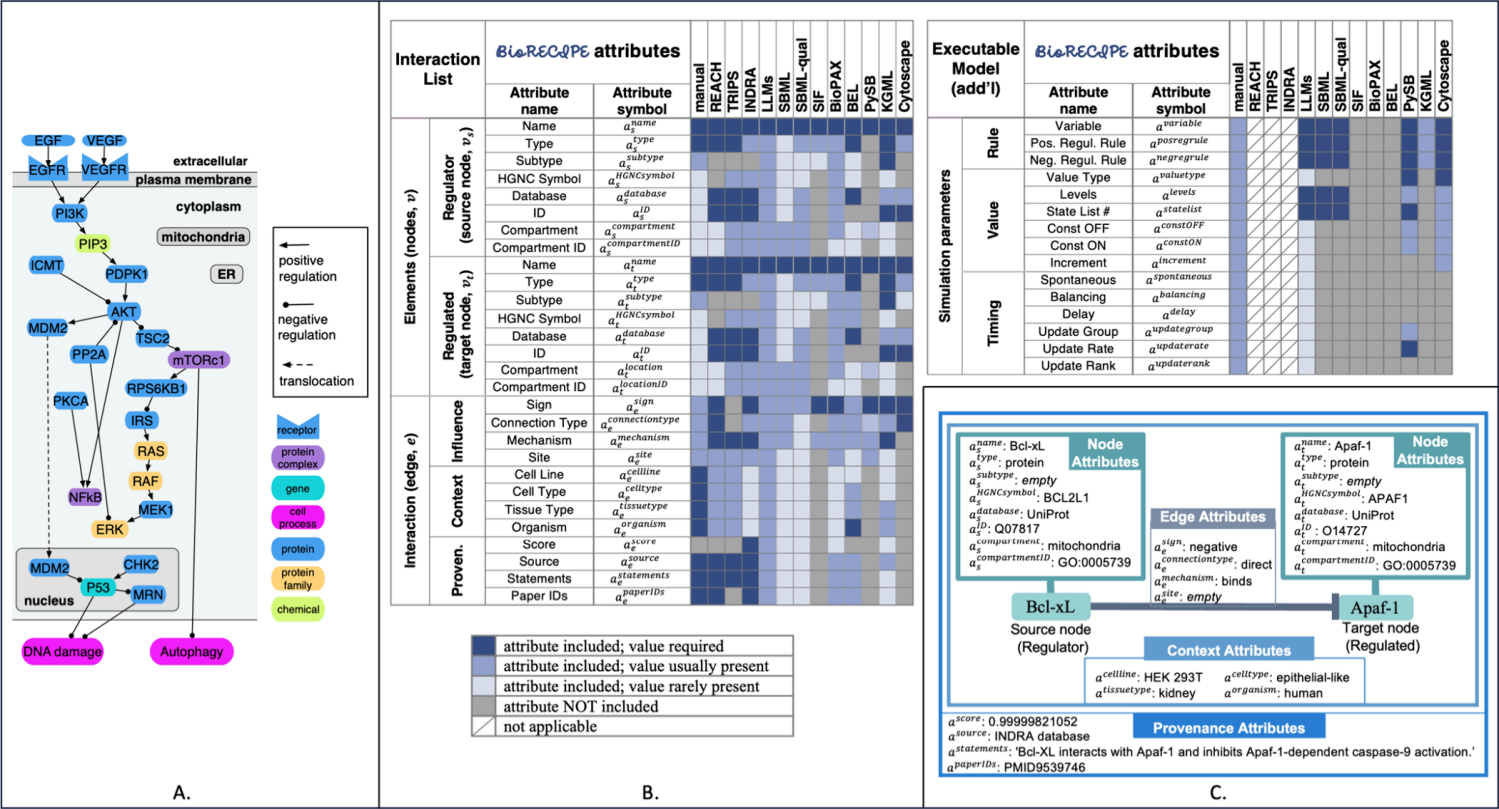
Examples: (A) Pathways, cell compartments, element types, and interactions that can be represented with BioRECIPE. (B) The list of all attributes used by BioRECIPE in Interaction List and Executable Model formats and a summary of whether these attributes are included and required by other formats and tools. (C) An example interaction and interaction attributes that are included in the BioRECIPE’s Interaction List format.

Systems biology and synthetic biology benefit from collaborations between biologists, computer scientists and engineers, therefore an easily readable, standardized representation of the complex events of cell signaling and gene regulatory networks (GRNs) (Figure 1A) is needed for sharing of information. A standardized format creates consistency, accuracy, and reproducibility, and makes models findable, accessible, interoperable, and reusable (FAIR principles).^1^

One common standard representation format is the Systems Biology Markup Language (SBML),^2^ which is based on XML and is therefore machine-readable. SBML uses modules to represent various components within a biological system such as reactions, species, and compartments, and it supports analysis via ordinary differential equations, stochastic simulation algorithm,^3^ or the reaction rule-based approach (e.g., BioNetGen).^4^ SBML format also allows for a significant amount of user annotation providing standardized representation of starting conditions, context, metadata, and literature sources. Cell Markup Language (CellML)^5^ is another XML-based standard representation format and a modeling framework used for executable models. These two formats differ in scope- CellML is ideal for detailed models of molecular interactions as it requires kinetic parameters for each interaction, while SBML is more suitable for modeling cell signaling pathways and networks. However, both SBML and CellML are not easily interpreted by life scientists without previous exposure to XML.

The Biological Pathway Exchange (BioPAX)^6^ provides another standardized format to represent molecular interactions in a signaling pathway. BioPAX supports three levels of representation, with each level offering increased complexity and detail. A simpler representation format, the Biological Expression Language (BEL),^7^ represents causal, correlative, and associative relationships between biological entities as a triplet statement. Similarly, the INDRA database^8^ represents causal relationships between entities in biomedical literature as statements with more detail than BEL. Some interaction and pathway databases may use their own representation format, for example, the KEGG Markup Language (KGML)^9^ was created for storing and standardizing models in the KEGG database.

Model representation formats rely on pre-existing ontologies to standardize individual biological entities and represent biological models. OBO (Open Biological and Biomedical Ontologies) is widely used to represent structured ontologies and controlled vocabularies, including the Gene Ontology (GO) Resource.^10^ It is human-readable and allows for the definition of classes, properties, and relationships between terms, making it suitable for standardizing biological models. While not strictly reserved for biological modeling, Web Ontology Language (OWL) is a more powerful and expressive language for creating ontologies. It uses formal logic to create semantic networks and is particularly useful for capturing complex relationships and reasoning in biological models in BioPAX format.

While powerful and flexible for machines, existing formats are difficult for humans to interact with. There is still a need for a standardized format that may be used for both static and executable models, is both human and machine-readable, can incorporate diverse annotations as well as data, and has translators for seamless conversion into other commonly used representation formats. To address this need, here we present the BioRECIPE representation format, which is interoperable with existing interaction, pathway, and model representation formats, and while it utilizes standardized ontologies, it is not dependent on any one ontology.

## Results

**Figure 2:**
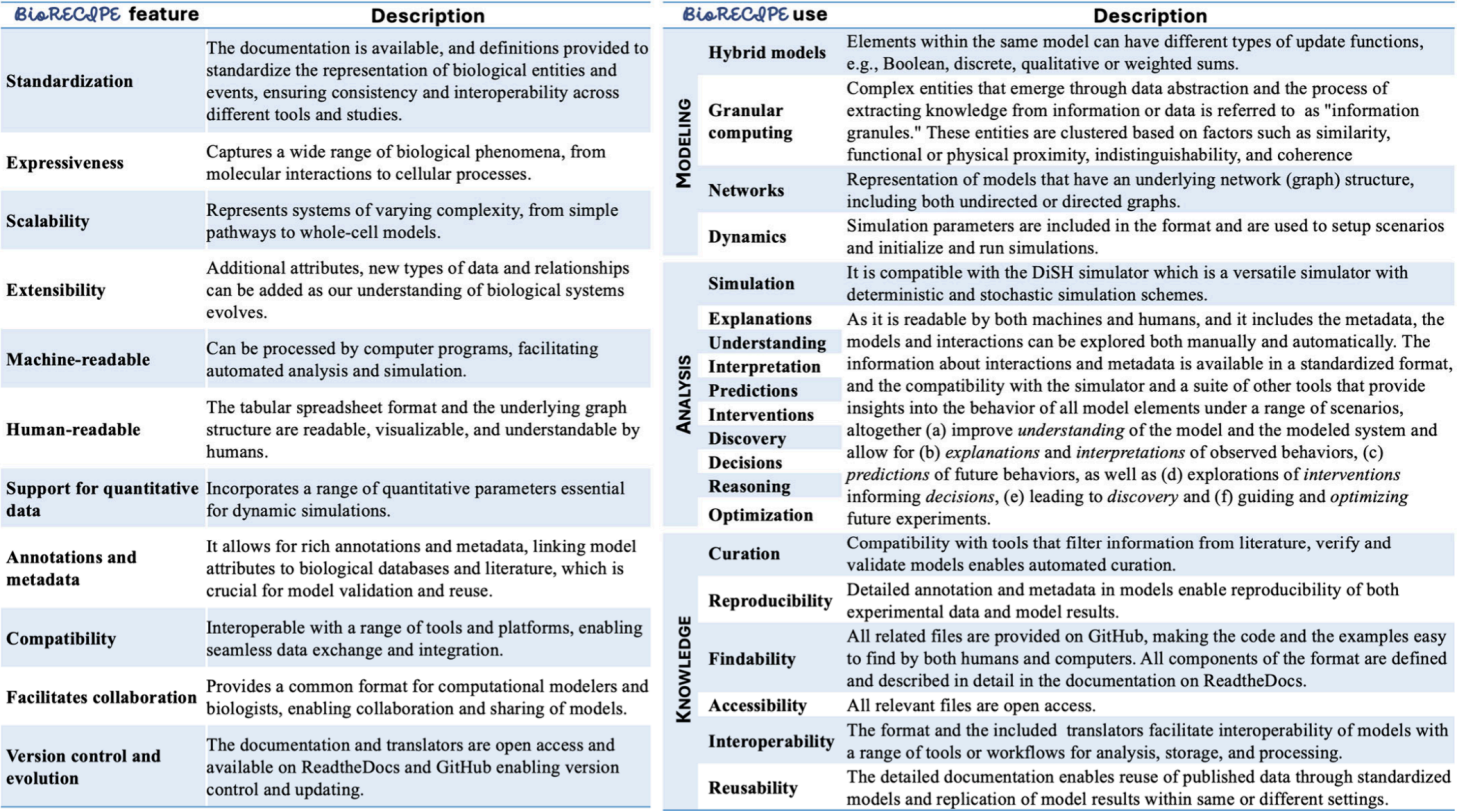
Description of BioRECIPE features and types of models that can be represented with BioRECIPE, model analysis that can be conducted on these models, and the descriptions of how BioRECIPE satisfies the FAIR principles.

BioRECIPE is a tabular representation format, typically written in a spreadsheet file type, which is simple for humans to use, facilitates knowledge sharing and aligns with FAIR principles (Figure 2). Spreadsheets have been used in life science domains as inputs for plasmid annotation packages,^11^ metabolic network modeling,^12^ and in synthetic biology.^13^ The BioRECIPE format can be used by both computational modelers and biology experts to create and modify Interaction List and Executable Model files. It also has a formal structure that can be read, created, updated, and output by computer programs. The detailed BioRECIPE documentation is available as ReadtheDocs pages,^14^ with instructions for creating Interaction Lists and Executable Models in this format.

In BioRECIPE, interactions are represented using the *event-based* Interaction List spreadsheet format, where each biological event is assigned one row, and the columns correspond to attributes of the event participants and the interaction between them. The attributes are selected and organized to allow for detailed curation and an extendable representation of interactions. An example biological interaction, represented as a directed signed edge between two nodes, including node, edge, context, and provenance attributes is illustrated in Figure 1C. More examples are included in the Supporting Information, ReadtheDocs documentation,^14^ and GitHub repository.^15^

The BioRECIPE format also provides representation of the static graph structure of models and attributes necessary to study the dynamics, usually through simulations. These executable models are represented in the BioRECIPE format using the *element-based* approach where each element in a model is assigned a row in the model spreadsheet, combining multiple interactions in which the element is the target of the influence. Element update functions are written using a simple notation in the BioRECIPE format, which supports discrete functions (min, max, weighted sum, Boolean), spontaneous increase and decrease of elements, different element update rates and types of regulation (positive, negative, highest level, weighted) and can be used under deterministic or stochastic simulation approaches.

Interactions and models written in the BioRECIPE format can be used by modelers, curators, and tool developers, and with a range of different tools that filter and classify interactions and automatically assemble and analyze models. The tools either use BioRECIPE directly or by translating interactions and models to and from other formats (Supporting Information). Interactions can be converted to the BioRECIPE format from the output of natural language processing tools or from interaction and pathway databases. Models can be converted from other representation formats and model databases. The BioRECIPE GitHub repository includes translators with instructions how to run them, examples of input and output files, and interactive Jupyter notebooks to guide the use of the BioRECIPE format and translators.^15^

## Conclusions

The BioRECIPE representation format is a valuable tool for systems and synthetic biology that enables comprehensive model curation by both humans and machines. Cellular signaling pathways and GRNs use the same components, in different combinations that are context specific, and therefore, interaction details are crucial to modeling accuracy. Automated readers are improving, however often fail to capture these details. BioRECIPE allows for all key element and interaction attributes to be included, as well as attributes for simulation, making it compatible with many existing interaction, pathway, and model databases, and with a range of tools for extraction of interaction information, model curation, simulation, and analysis. This interoperability ensures that researchers can seamlessly integrate BioRECIPE into their existing workflows. Future directions include additional functionality to translate between other existing formats (e.g., SBOL^16^), or integration of the BioRECIPE representation format as input to model curation and storage platforms (e.g., CellCollective,^17^ NDEx^18^). These platforms play a crucial role in managing and disseminating computational models, and the integration with BioRECIPE could further streamline the process of model sharing and collaboration within the scientific community.

## Supporting Information Available

Links to BioRECIPE resources (ReadtheDocs, GitHub folders, Jupyter notebooks) and example containing attribute values extracted from sentences and a summary of standard database tools, formats, and translators for BioRECIPE format translation and conversion (PDF)
